# Role of Poly Adenosine Diphosphate Ribose Polymerase Inhibitors in Advanced Stage Ovarian Cancer

**DOI:** 10.7759/cureus.2685

**Published:** 2018-05-24

**Authors:** Ena Arora, Muhammad Masab, Vishal Jindal, Iqra Riaz, Sorab Gupta, Gabor Varadi

**Affiliations:** 1 Obstetrics and Gynecology, Civil Hospital Chandigarh, Chandigarh, IND; 2 Internal Medicine, Albert Einstein Medical Center, New York, USA; 3 Internal Medicine, St. Vincent Hospital Worcester, Worcester, USA; 4 Department of Medicine, Mayo Hospital, King Edward Medical University, Lahore, Pakistan; 5 Hematology and Oncology, Albert Einstein Medical Center, New York, USA

**Keywords:** ovarian cancer, parp inhibitors, olaparib, rucaparib, niraparib, poly (adenosine diphosphate [adp]-ribose) polymerase (parp)

## Abstract

Ovarian cancer is one of the leading causes of death from gynecologic cancers. In this present era of cancer treatment, therapeutic options for patients with advanced or recurrent ovarian cancer are limited. The present standard of care treatment for advanced ovarian cancer is a platinum-based doublet chemotherapy (paclitaxel and carboplatin with or without bevacizumab) after a maximum attempt of surgical cytoreduction. However, there are no promising options for the management of patients with ovarian cancer refractory to the platinum-based chemotherapy. Therefore, newer, safe, and more effective treatment modalities are required for patients with advanced or recurrent ovarian cancer. Poly(adenosine diphosphate [ADP]-ribose) polymerase (PARP) inhibitors have shown an impressive safety profile and anti-tumor efficacy in patients with breast cancer 1 and 2 (BRCA1 and BRCA2) gene-mutated ovarian cancer who were previously treated with the standard of care chemotherapy. We have done a detailed review of the literature to emphasize the role of PARP inhibitors in the treatment of advanced or relapsed ovarian cancer.

## Introduction and background

Ovarian cancer is one of the leading causes of death from gynecologic cancers. It is the fifth most common cause of cancer-related deaths amongst women of the United States. In 2018, epidemiological studies have shown that there will be around 22,240 new diagnoses and 14,070 deaths from ovarian cancer in the United States. The five-year survival rate from ovarian cancer is up to 46.5%. Also, the cure rate is around 40% [1-2]. The median age of diagnosis is 63 years, and the majority of women (70%) have advanced disease at presentation [1-2].

Some of the main risk factors associated with ovarian cancers are late pregnancy, nulliparous women, hormone replacement therapy, pelvic inflammatory disease, endometriosis, and genetic mutations [3-5]. Patients having two or more first-degree relatives with ovarian cancer usually have an early onset of the disease, especially with the breast cancer gene 1 and 2 (BRCA1 and BRCA2) germline mutations or Lynch syndrome [6-18].

Human cells continue to develop mutations throughout their life that can lead to cell death if the damage is extensive. Moreover, sometimes mutations can also allow a cell to acquire a malignant potential. There are different deoxyribonucleic acid (DNA) repair mechanisms to neutralize these mutations. Some of these repair pathways are nucleotide-excision, base-excision, homologous recombination, mismatch repair, and telomerase metabolism.

Poly(adenosine diphosphate [ADP]-ribose) polymerases (PARPs) are a group of enzymes that carry on the modification of post-translational proteins. PARP-1 and PARP-2 are the main subtypes of the PARP family, that are implicated in the base-excision repair (BER) of single-stranded breaks (SSBs) [19-20]. PARPs also play an important role in the homologous recombination (HR) pathway. It identifies the disrupted replication forks and works with the DNA protein kinase complex involved in non-homologous end-joining (NHEJ) [21]. BRCA1 and BRCA2 have a significant role in the double-stranded repair through the HR pathway [22]. Cells with mutations in BRCA1 or BRCA2 lose the function to repair double-stranded breaks and thus, utilize NHEJ for DNA repair. PARP inhibition is not deadly to cells without the BRCA mutation because of a normal working HR pathway. As cells with a BRCA mutation are deficient in the HR pathway, the loss of PARP activity in such cells will result in cell death. Synthetic lethality was the term coined for this mechanism, and it led to further research of the PAPR inhibition [23-24]. 

PARP inhibitors work by attaching to the nicotinamide adenine dinucleotide (NAD+) binding site of the PARP molecule, thereby inhibiting its function. Various studies have shown that BRCA-mutated cells are significantly affected by PARP inhibition [25-26].

## Review

Olaparib 

Olaparib is a PARP inhibitor approved for the management of ovarian cancer, both for initial as well as maintenance treatment. It showed impactful response rates in the BRCA1 or BRCA2-mutated platinum-sensitive ovarian cancer patients [27-32].

SOLO2/ENGOT-OV21A was a clinical trial evaluating olaparib as a maintenance treatment in BRCA 1/2 platinum-sensitive, relapsed ovarian cancer patients who had received two or more lines of chemotherapy (registered with ClinicalTrials.gov, #NCT01874353). It included patients with high-grade serous, endometrial, primary peritoneal, or fallopian tube cancer. Two hundred and ninety-five eligible patients were randomly assigned to receive olaparib or a placebo treatment. This trial showed a longer median progression-free survival with olaparib, which was significant (19.1 months compared with 5.5 months for placebo). In the olaparib group, 35 (18%) patients showed serious adverse events as compared to the placebo group (8%). The common adverse events of Grade 3 or worse severity were anemia (19% in the olaparib group vs 2% in the placebo group), fatigue (4% in olaparib group vs 2% in placebo group), and neutropenia (5% in olaparib group vs 4% in placebo group) [33].

Another randomized, double-blinded, placebo-controlled, Phase 2 study (registered with ClinicalTrials.gov, #NCT00494442) evaluated olaparib in the maintenance settings in patients with platinum-sensitive, relapsed, high-grade serous ovarian cancer who had received two or more platinum-based regimens with a partial or complete response [32]. Two hundred and sixty-five patients were enrolled, and regardless of BRCA status, they were randomly assigned to receive 400 mg twice daily of olaparib vs placebo. This study showed a statistically significant improvement in progression-free survival (PFS) in patients treated with olaparib (8.4 months) as compared to placebo (4.8 months). The adverse events observed were mainly Grade 1 or 2 in the olaparib group and these were nausea (68%), fatigue (49%), vomiting (32%), and anemia (17%).

Based on these clinical trials, the Food and Drug Administration (FDA) and the National Comprehensive Cancer Network (NCCN) guidelines recommended olaparib to be used as a maintenance therapy for women with advanced stage ovarian cancer who have received two or more lines of chemotherapy.

The FDA also approved olaparib as a single agent for the treatment of advanced ovarian cancer patients with germline BRCA1 or BRCA2 mutation who had received three or more prior lines of chemotherapy. This approval was based on the results of a single arm Phase 2 study, that enrolled 298 patients with solid tumors and BRCA1 or BRCA2 mutations [34]. All patients had received prior platinum-containing chemotherapy and were considered to be platinum-resistant or not suitable for further platinum therapy. Of the 193 patients in the ovarian cancer cohort, 178 had ovarian cancer, four had fallopian tube cancer, and 11 had primary peritoneal cancer. All patients were pretreated with chemotherapy with a mean number of prior regimens up to 4.3. Response rates were 31.1% in patients with ovarian cancer. Patients had a median PFS of seven months, a median overall survival (OS) of 16.6 months, and one year OS of 64.4% [34]

Rucaparib

Rucaparib is an oral PARP inhibitor used in advanced (Stage III-IV) ovarian cancer. A Phase 2 trial (ARIEL-2) (registered with ClinicalTrials.gov, #NCT01891344) evaluated the safety and efficacy of rucaparib in the treatment of relapsed platinum-sensitive ovarian cancers in patients who had received at least two prior lines of platinum-based chemotherapy. A total of 192 patients were divided into three subgroups: BRCA mutant, BRCA wild-type with high loss of heterogeneity (LOH) defined as 14% or more genomic LOH (LOH high group), or BRCA wild-type with less than 14% genomic LOH (LOH low group). Rucaparib was given continuously at a dose of 600 mg twice per day (28-day cycle) until the patients had disease progression or developed an adverse event. The primary endpoint was median progression-free survival (PFS). The median PFS was 12.8 months in the BRCA mutant group, 5.7 months in the LOH high group, and 5.2 months in the LOH low group. Patients in the LOH high group had improved responses to rucaparib (39% compared to 69% for the BRCA mutation subgroup and 11% for the BRCA wild-type, LOH low patients) [9]. Common Grade 3 or more adverse events observed with the drug were anemia (22%) and elevations in alanine aminotransferase or aspartate aminotransferase (12%) [35]. 

The results of this trial showed the efficacy of rucaparib in BRCA-mutant patients and also suggested that the assessment of genomic LOH can help to identify platinum-sensitive ovarian cancer patients with BRCA wild-type who can respond to rucaparib.

Niraparib

Niraparib is an oral PARP inhibitor which was investigated in a randomized, double-blind, Phase 3 trial (NOVA) to study the clinical efficacy of maintenance niraparib in platinum-sensitive, recurrent ovarian cancer patients who had received at least two previous lines of platinum-based treatment (registered with ClinicalTrials.gov, #NCT01847274) [36]. This study enrolled 597 patients with a BRCA1/2 germline mutation, as well as the BRCA wild-type. BRCA wild-type patients were further stratified on the basis of homologous recombination deficiency (HRD). Patients were randomly allocated to receive niraparib (300 mg) or placebo once daily. The primary endpoint was PFS.

Patients in the niraparib group had a significantly longer median PFS as compared to the placebo group (21.0 months vs 5.5 months, respectively) in the BRCA-mutant group. The median PFS was also improved in the BRCA wild-type group with positive HRD who received niraparib compared to placebo (12.9 months vs. 3.8 months, respectively). The median PFS for niraparib in patients with overall BRCA wild-type group, irrespective of HRD, was 9.3 months vs. 3.9 months for the placebo. The most common Grade 3 or 4 adverse events in the niraparib group were thrombocytopenia (33.8%), anemia (25.3%), and neutropenia (19.6%) [36]. 

The NOVA trial results demonstrated that median PFS for patients with platinum-sensitive, recurrent ovarian cancer was significantly longer in patients who received niraparib as compared to placebo, irrespective of the BRCA mutation or HRD status [36]. 

## Conclusions

In summary, PARP inhibitors demonstrate a very promising safety profile and anti-tumor efficacy in patients with BRCA1 or BRCA2-mutated platinum-sensitive ovarian cancer patients in both treatment and maintenance settings. The recent clinical studies are also encouraging to illustrate its role in some platinum refractory and BRCA wild-type ovarian cancer patients. More studies are needed for a better understanding of the use of PARP inhibitors in ovarian cancer. 
